# Supplementary material for: FhMYB108 Regulates the Expression of Linalool Synthase Gene in Freesia hybrida and Arabidopsis
Source: Biology (Basel). 2024 Jul 23;13(8):556. doi: 10.3390/biology13080556 (PMC11352005; doi:10.3390/biology13080556)
Supplement: Supplementary file 1 [file biology-13-00556-s001.zip › biology-3081056-supplementary.pdf]

Table S1. Primers used in this study.

Function description	Gene names	Forward（5'-3'）	Reverse（5'-3'）
Gene clone	FhMYB21L1	ATGGACAAAGGCATAAATAGC	TTAGTCCCCATTGAGTGGC
	FhMYB21L2	ATGGACAAGAGGATAAATAGTG	TTACCCCCCAAAAATTGG
	FhMYB108	ATGACGTCGACGCCTACGTCGAGC	TTACAGCTGCTGCTGAATTAGCCA
pUC19-HA/GD	FhMYB108	GCTCTAGAATGACGTCGACGCCTAC	CGAGCTCTTACAGCTGCTGCTGAATTAG
Transient expression vector	AtMYC2	CAACATATGATGACTGATTACCGGCTACAA	CAAATCGATTTAACCGATTTTTGAAATCAAA
	Fh_{Pro}:TPS1(0-1241)	GCTGCAGTTTCTTGGCGGTCCGTAGATTAG	CGAGCTCTATAATATGTTTCTCTTTAGCTTTT
	Fh_{Pro}:TPS1(0-1016)	GCTGCAGACGGTGCTTTAATAGTAGAGTAC	CGAGCTCTATAATATGTTTCTCTTTAGCTTTT
	Fh_{Pro}:TPS1(0-914)	GCTGCAGATCTTTTGTTTGGTGCTACG	CGAGCTCTATAATATGTTTCTCTTTAGCTTTT
	Fh_{Pro}:TPS1(0-472)	GCTGCAGTACCAGTGGCGGATTTAG	CGAGCTCTATAATATGTTTCTCTTTAGCTTTT
	Fh_{Pro}:TPS1(0-214)	GCTGCAGATCGCATGATCATATCTATTG	CGAGCTCTATAATATGTTTCTCTTTAGCTTTT
	At_{Pro}：TPS11	GCTGCAGCACAATCTGAGGTCGGCGGTC	CGAGCTCAACGAAGAAGAGAAGAAAAAA
	At_{Pro}：TPS14	GCTGCAGAAATAGTAACCGAAAAATCTGG	CGAGCTCGATGTATGAACTTAAGTTTTGTT
	At_{Pro}：TPS21	GCTGCAGAAAAATGTCGTCCCGTGGCCG	CGAGCTCGCTAAAAATTTCTGGTAGAAGT

pUC19-GFP	**FhMYB108-GFP**	**GCTCTAGAATGACGTCGACGCCTAC**	**CCATCGATCAGCTGCTGCTGAATTAG**
Transient expression vector	**FhMYB108-GFPN**	**CGCCATATGATGACGTCGACGCCTAC**	**CCATCGATTTACAGCTGCTGCTGAATT**
	**AtMYC2-GFPC**	**CGCCATATGATGACTGATTACCGGCTACA**	**CCATCGATTTAACCGATTTTTGAAATCA**
pBI121	**FhMYB108**	**GCTCTAGAATGACGTCGACGCCTAC**	**CGAGCTCTTACAGCTGCTGCTGAATTAG**
**Stable transfection vector**			
**qRT-PCR**	**FhMYB108**	**GGAGGACGGATAACGAGATAAAG**	**GACGTGGCATCCATAGGTATC**
	**AtTPS11**	**CGAGCCCACCACATATTTCA**	**GTGGTCCTCTTCGTGTTCATAG**
	**AtTPS14**	**ACGTCCAAGAGGTTCACAAG**	**TCTCCGCCACTCGTAATAGA**
	**AtTPS21**	**CGGAACTGAGACGTTCAAAGAG**	**AGACACCAAGGCGACATAGA**
	**β-actin**	**GCTGAGAGATTCAGATGCCCA**	**GTGGATTCCAGCAGCTTCCAT**
